# Standard-Based Port-Sharing Workflow for Simultaneous Robot-Assisted Distal Gastrectomy and Right-Sided Colectomy: A Case Report

**DOI:** 10.70352/scrj.cr.26-0164

**Published:** 2026-06-09

**Authors:** Sho Ueda, Shintaro Yamazaki, Miyuki Takahashi, Mariko Masubuchi, Aina Kunitomo, Kentaro Shinohara, Tatsuki Matsumura, Yasuyuki Fukami, Kazuhiko Wakabayashi, Tsuyoshi Sano, Yutaka Ito

**Affiliations:** 1 Department of Gastroenterological and Mammary Surgery, National Hospital Organization Disaster Medical Center, Tachikawa, Tokyo, Japan; 2 Department of Gastroenterological Surgery, Aichi Medical University, Nagakute, Aichi, Japan

**Keywords:** colorectal neoplasms, robotic surgical procedures, stomach neoplasms

## Abstract

**INTRODUCTION:**

Synchronous gastric and colorectal cancers occur in approximately 4% of patients. Although simultaneous resection is common in laparoscopic surgery, robot-assisted simultaneous procedures are less frequently reported because of logistical constraints in multi-quadrant targeting and port geometry. Herein, we describe a standardized and reproducible workflow using the da Vinci Xi system (Intuitive Surgical, Sunnyvale, CA, USA) that focuses on strategic port sharing and planned redocking.

**CASE PRESENTATION:**

An 86-year-old man presented with gastric cancer (cT3N0M0, Stage II), ascending colon cancer (cT2N0M0, Stage I), and symptomatic cholelithiasis. The procedure was performed using a da Vinci Xi system with single-direction docking from the patient’s left side. A 5-cm mini-laparotomy and 6 robotic ports were used. To enhance reproducibility, the integrated layout was designed to align with our institutional standard setups (National Hospital Organization Disaster Medical Center) for isolated distal gastrectomy and right-sided colon resection. Most ports were shared, with only the R1 working arm relocated between phases to optimize the triangulation for each target. The surgical sequence was strategically planned to minimize table-tilt transitions, starting with right-sided colectomy in the Trendelenburg position, followed by a single transition to the reverse Trendelenburg position for D3 lymphadenectomy and the subsequent gastric phase. After distal gastrectomy, cholecystectomy was performed during optimal upper abdominal exposure. The surgery was successfully completed by a small team of 1 console surgeon and 1 bedside assistant. The operative and console times were 390 and 350 min, respectively, with 20 mL of blood loss. Final pathology showed R0 resection for both cancers (gastric: pT2N1M0; colon: pT1N0M0). The patient’s postoperative course was favorable despite a Clavien–Dindo grade II serous leakage, which was resolved with antibiotics.

**CONCLUSIONS:**

A standardized workflow featuring fixed docking, planned targeting, and port sharing may facilitate robot-assisted simultaneous resection in selected patients. By combining standard institutional setups, the approach may help streamline dual-quadrant logistics while preserving ergonomic triangulation; however, further case accumulation is needed.

## Abbreviation

Xi
: da Vinci Xi surgical system

## INTRODUCTION

Synchronous gastric and colorectal cancers occur in approximately 4% of patients.^1)^ Although simultaneous resection is common in open and laparoscopic surgery, robot-assisted simultaneous procedures are less frequently reported because of multi-quadrant constraints in targeting, redocking, and port geometry.^2–4)^ Herein, we describe a standardized workflow for combined distal gastrectomy and right-sided colectomy using da Vinci Xi (Intuitive Surgical, Sunnyvale, CA, USA), featuring strategic port sharing and a planned targeting/redocking strategy.

## CASE PRESENTATION

### Patient and indication

An 86-year-old man presented with gastric cancer (cT3N0M0, Stage II; Union for International Cancer Control TNM Classification of Malignant Tumours, 8th edition),^5)^ ascending colon cancer (cT2N0M0, Stage I), and symptomatic cholelithiasis. The patient had a performance status of 0 and was considered fit for surgery.

### Docking, access, and port placement

The Xi (Intuitive Surgical) cart was docked on the left side of the patient at a slight cephalad angle for both procedures. A 5-cm upper abdominal mini-laparotomy was created and fitted with a wound protector (Applied Medical, Rancho Santa Margarita, CA, USA) and an AirSeal access port (ConMed, Largo, FL, USA). Pneumoperitoneum was maintained at a pressure of 10 mmHg.

**Fig. 1:**
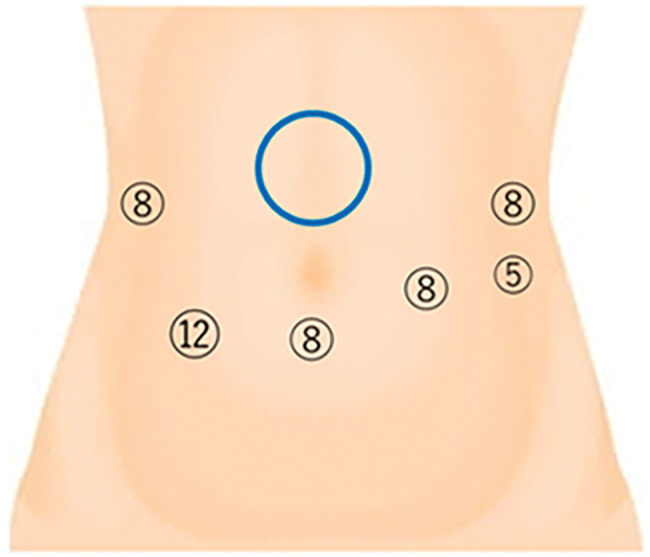
Integrated port placement for simultaneous robot-assisted gastric and colon surgery (using the da Vinci Xi system). The blue circle indicates a 5-cm upper abdominal mini-laparotomy. Numbered circles indicate port size (mm). Ports were planned for extensive sharing across phases with minimal relocation. da Vinci Xi system, Intuitive Surgical, Sunnyvale, CA, USA

The ports were placed as follows: an infraumbilical 8-mm camera port (R2), a right lower abdominal 12-mm robotic port (R1 for right-sided colectomy, which was reused as the assistant port for gastrectomy), a right lateral 8-mm robotic port (R1 for gastrectomy only), a left lateral 8-mm robotic port (R4), an 8-mm robotic port midway between R2 and R4 (R3), and a 5-mm assistant port lateral to the midpoint between R3 and R4 (used during the right-sided colon phase only). Reusing the 12-mm port as an assistant port preserved the ergonomic triangulation of the surgeon while ensuring adequate assistant access for gastric retraction. The procedure involved a mini-laparotomy with 6 ports (**Fig. 1**).

**Fig. 2:**
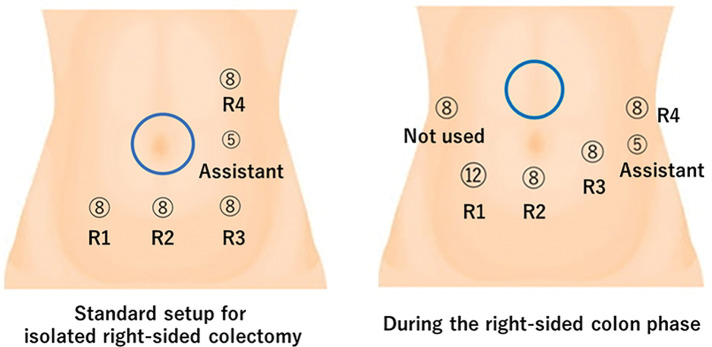
Port utilization during the right-sided colon phase in the integrated workflow. The right lower abdominal 12-mm robotic port served as R1, the 5-mm assistant port was used only in this phase, and the right lateral 8-mm robotic port was not used. The camera was placed in R2.

**Fig. 3:**
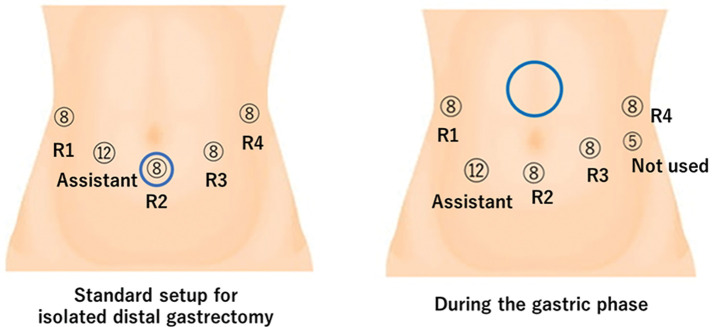
Port utilization during the gastric phase in the integrated workflow. After retargeting to the diaphragmatic crus, the R1 working arm was moved from the right lower abdominal 12-mm port to the right lateral 8-mm port to optimize triangulation. The right lower abdominal 12-mm port was then reused as the assistant port.

To enhance reproducibility, the layout was designed to align with our institutional standard setup (National Hospital Organization Disaster Medical Center) for isolated procedures, with limited role-switching and R1 relocation (**Figs. 2** and **3**).

### Positioning, targeting, and redocking

**Fig. 4:**
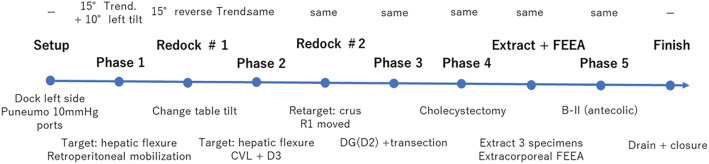
Simplified timeline of table tilt, targeting, and planned redocking. The workflow included 2 planned redocking events aligned with phase transitions, followed by specimen extraction, extracorporeal ileocolic anastomosis, and intracorporeal antecolic B-II reconstruction. B-II, Billroth II; CVL, central vascular ligation; DG, distal gastrectomy; FEEA, functional end-to-end anastomosis; Trend., Trendelenburg

Surgery was initiated at a 15° Trendelenburg position with a 10° left tilt, targeting the hepatic flexure. The table was then changed to a 15° reverse Trendelenburg position for central vascular ligation (1st redocking). While maintaining this position, the targeting was reset to the diaphragmatic crus during the gastric phase. The R1 arm was relocated from the 12-mm port to the right lateral 8-mm port to optimize triangulation (2nd redocking) (**Fig. 4**).

### Operative sequence and robotic instruments

Arm 1 used fenestrated bipolar forceps and Arm 4 used a tip-up fenestrated grasper. In Arm 3, monopolar curved scissors and a vessel sealer extension were used for the right-sided colectomy, and Maryland bipolar forceps were added for gastrectomy.

The sequence was as follows (**Supplementary Video 1**):

1. Right-sided colectomy (mobilization and D3 lymphadenectomy).
2. Distal gastrectomy (D2 lymphadenectomy and gastric transection).
3. Cholecystectomy (during optimal upper abdominal exposure).
4. Extracorporeal functional end-to-end ileocolic anastomosis via mini-laparotomy (Echelon 60, blue; Ethicon Endo-Surgery, Cincinnati, OH, USA).
5. Intracorporeal antecolic Billroth II reconstruction (side-to-side gastrojejunostomy; Echelon 60, blue).

### Outcomes, pathology, and postoperative course

The operative and console times were 390 and 350 min, respectively, with 20 mL of blood loss. The 40-min difference between operative and console time largely reflected table repositioning/redocking and extracorporeal reconstruction, which helps illustrate the logistical components of multi-quadrant surgery in this case. Final pathology confirmed R0 resection for both gastric cancer (pT2N1M0, Stage IIA; 36 nodes) and ascending colon cancer (pT1N0M0, Stage I; 15 nodes). The patient was discharged on POD 9 and readmitted on POD 12 for a Clavien–Dindo grade II serous leakage at the D2 bed, which was successfully treated with antibiotics.^6)^

## DISCUSSION

To the best of our knowledge, only 3 reports have described robot-assisted simultaneous resection for synchronous gastric and colorectal cancers.^2–4)^ In those reports, the emphasis was placed on maximizing port sharing to reduce the number of skin incisions.^2,3)^ In contrast, our “standard-based” approach involves designing an integrated layout that closely follows our institutional standard port setups for each isolated procedure (distal gastrectomy and right-sided colectomy), while allowing only limited role switching and relocation of the R1 arm. This approach aims to improve reproducibility by building on familiar, routine geometries rather than requiring case-specific port arrangements (**Figs. 2** and **3**).

Although the workflow required 6 ports and a mini-laparotomy for specimen extraction and extracorporeal ileocolic anastomosis, the mini-laparotomy was used for both extraction and reconstruction, and the port layout was intentionally kept close to our standard robotic setups. Thus, the approach aims to balance operability and invasiveness rather than to minimize port number per se.

However, the integrated layout may have practical limitations in a subset of patients. In our 86-year-old patient, the terminal ileum was physiologically adherent to the caudal retroperitoneum, and dissection extended slightly more caudally than in a typical right-sided colectomy, resulting in transient range-of-motion restriction of Arm 1. When restriction occurred, we temporarily avoided using Arm 1, maintained exposure with Arm 4 and the bedside assistant, and completed adhesion takedown using scissors on Arm 3; thereafter, Arm 1 could be used smoothly for the remainder of mobilization. Because caudal adhesions of the terminal ileum are not uncommon, such arm restriction may occur in similar cases. A potential modification would be to place the R1 port slightly more caudally to improve reach for caudal dissection; however, this may compromise assistant access during the gastric phase. Further experience is needed to optimize this balance.

In this report, “efficiency” refers to a workflow design that minimizes non-operative time related to multi-quadrant logistics (e.g., table-position changes, targeting, and redocking), whereas “reproducibility” refers to the use of a standard-based port geometry that can be replicated by teams already familiar with each single procedure. Because this is a single case, these concepts should be interpreted as feasibility-oriented rather than as evidence of superiority.

The surgical sequence was strategically designed to minimize logistic redundancy and to optimize the physiological management in an 86-year-old patient. A critical challenge in multi-quadrant robotic surgery is the time-consuming process of repositioning the patient cart and the operating table. By initiating the procedure with right-sided colectomy in the Trendelenburg position and transitioning only once to the reverse Trendelenburg position for the subsequent gastric phase, we consolidated the major logistical adjustments into a single event. Reversing this sequence and performing gastrectomy first would have necessitated multiple postural changes between the Trendelenburg and reverse Trendelenburg positions for retroperitoneal mobilization and lymphadenectomy, respectively. Reported operative times for robot-assisted simultaneous gastrectomy and colorectal resection vary widely. In 2 reports of combined gastrectomy and right-sided colectomy, operative time was 640 min in 1 case and 380 min in the other.^2,3)^ In a report of combined gastrectomy and rectal resection, the operative times were reported separately for each component (281 min for gastrectomy and 327 min for rectal resection).^4)^ Our operative time (390 min) appears to be within this reported range. However, direct comparison is difficult because of differences in procedures, reconstruction methods, additional procedures (e.g., cholecystectomy), and definitions of operative time. Given the limited number of reports, our workflow may be feasible without an excessive increase in operative time, but further case accumulation is needed.

The robotic platform offers distinct advantages for multi-quadrant surgery. A stable, high-definition 3D view and EndoWrist technology (Intuitive Surgical) were instrumental in performing precise lymphadenectomy in the narrow fields of D2 (gastric) and D3 (colonic) dissections. Furthermore, the “fixed-point” technology of the robotic system, known as the remote center of motion, minimizes abdominal wall trauma.^7)^ In conventional laparoscopy, the wide-range movements required for multi-quadrant access often exert significant stress on port sites owing to the fulcrum effect. In contrast, the robotic arm maintains a precise pivot point at the incision, reducing shear forces on the abdominal wall and potentially improving postoperative recovery, even in extensive combined resections.^8)^

The ergonomic benefits of the robotic console also played a vital role in maintaining surgical quality. Unlike laparoscopic surgery, in which the primary surgeon must often change standing positions or endure physical strain during prolonged multi-organ procedures, the console allowed for a consistent seated position. This markedly reduced physical fatigue, ensuring that the surgeon’s precision remained high until the final reconstruction phase. Remarkably, this dual-quadrant surgery was completed by a team consisting of only 1 console surgeon and 1 bedside assistant. Manpower efficiency is a significant advantage in modern surgical practice, which is characterized by surgeon shortages and the need for standardized, labor-efficient workflows.

The postoperative serous leakage (Clavien–Dindo grade II) occurred at the gastric D2 dissection bed around station No. 6 and was resolved with antibiotics. Although a causal relationship cannot be established, several factors may have contributed, including the patient’s advanced age, the extent of lymphadenectomy, and the prolonged operative duration inherent to a combined multi-quadrant procedure. In addition, repeated table-position changes and redocking may increase fatigue and workflow complexity, thereby indirectly affecting meticulous hemostasis/lymphostasis. Because right-sided colectomy was performed concomitantly, the duodenum and pancreatic head were mobilized more extensively than in a standard gastrectomy alone, which may have increased oozing/lymphatic leakage around the No.6 dissection bed. Importantly, our port-sharing strategy and R1 relocation were planned to maintain standard triangulation during the upper abdominal phase (**Fig. 3**); nevertheless, surgeons should be aware that any deviation from routine ergonomics could theoretically affect precision during demanding dissections, such as the infrapyloric (No. 6) lymphadenectomy. Careful re-check of the dissection bed after redocking and before closure, and a low threshold for additional hemostatic/lymphostatic measures, are advisable when adopting this approach.

Regarding reconstruction, the choice of method should be individualized based on the patient’s age and the specific combination of procedures. Although the optimal reconstruction after distal gastrectomy remains debatable,^9,10)^ we selected an antecolic Billroth II gastrojejunostomy. This choice was based on its technical simplicity and minimized the risk of anastomotic tension or obstruction that might arise from postoperative duodenal or mesenteric edema following concurrent right-sided colectomy. In our 86-year-old patient, this straightforward reconstruction contributed to a successful oncological outcome (R0 resection) without major anastomotic complications.

This report is limited by its single-case nature. However, our standard-based workflow suggests that robot-assisted simultaneous resection can be feasible in selected patients. By aligning multi-quadrant strategies with routine institutional practices, the approach may help streamline logistics and improve reproducibility; however, further case accumulation is needed.

## CONCLUSIONS

A standardized workflow featuring fixed docking, planned targeting, and port sharing may facilitate robot-assisted simultaneous resection in selected patients. By combining standard institutional setups, this approach may help streamline dual-quadrant logistics while preserving ergonomic triangulation; however, further case accumulation is needed to evaluate efficiency and safety more objectively.

## SUPPLEMENTARY MATERIALS

Supplementary video 1 Robot-assisted simultaneous resection using a standardized port-sharing workflow (using the da Vinci Xi system [Intuitive Surgical]).
The video shows left-sided docking, phase-specific table tilt (15° Trendelenburg position with 10° left tilt to 15° reverse Trendelenburg position), targeting (from the hepatic flexure to the diaphragmatic crus), specimen extraction with extracorporeal functional end-to-end ileocolic anastomosis, and intracorporeal antecolic Billroth II reconstruction.

## DECLARATIONS

### Availability of data and materials

The datasets used and/or analyzed during the current study are available from the corresponding author on reasonable request.
